# Targeted Nanoparticles Harboring Jasmine-Oil-Entrapped Paclitaxel for Elimination of Lung Cancer Cells

**DOI:** 10.3390/ijms22031019

**Published:** 2021-01-20

**Authors:** Shira Engelberg, Yuexi Lin, Yehuda G. Assaraf, Yoav D. Livney

**Affiliations:** 1 The Laboratory of Biopolymers for Food and Health, Department of Biotechnology and Food Engineering, Technion—Israel Institute of Technology, Haifa 3200003, Israel; shira125@campus.technion.ac.il (S.E.); yuexi.lin@campus.technion.ac.il (Y.L.); 2 The Fred Wyszkowski Cancer Research Laboratory, Department of Biology, Technion—Israel Institute of Technology, Haifa 3200003, Israel

**Keywords:** lung cancer, targeted delivery, nanoparticles, jasmine oil, paclitaxel, coencapsulation, synergy

## Abstract

Selectively targeted drug delivery systems are preferable chemotherapeutic platforms, as they specifically deliver the drug cargo into tumor cells, while minimizing untoward toxic effects. However, these delivery systems suffer from insufficient encapsulation efficiency (EE), encapsulation capacity (EC), and premature drug release. Herein, we coencapsulated paclitaxel (PTX) and Jasmine oil (JO) within PEG-PCL nanoparticles (NPs), with an average diameter < 50 nm, selectively targeted to non-small cell lung cancer (NSCLC) cells, via S15-aptamer (APT) decoration. JO was selected as an “adhesive” oily core to enhance PTX entrapment, as JO and PTX share similar hydrophobicity and terpenoid structure. JO markedly enhanced EE of PTX from 23% to 87.8% and EC from 35 ± 6 to 74 ± 8 µg PTX/mg PEG-PCL. JO also markedly increased the residual amount of PTX after 69 h, from 18.3% to 65%. Moreover, PTX cytotoxicity against human NSCLC A549 cells was significantly enhanced due to the co-encapsulation with JO; the IC_50_ value for PTX encapsulated within JO-containing APT-NPs was 20-fold lower than that for APT-NPs lacking JO. Remarkably, JO-containing APT-NPs displayed a 6-fold more potent cell-killing, relatively to the free-drug. Collectively, these findings reveal a marked synergistic contribution of JO to the cytotoxic activity of APT-NP-based systems, for targeted PTX delivery against NSCLC, which may be readily applied to various hydrophobic chemotherapeutics.

## 1. Introduction

Lung cancer remains the leading cause of cancer-related death worldwide [1]. Non-small cell lung cancer (NSCLC) is the prevalent lung cancer type, responsible for over 85% of lung cancer cases [2]. Nowadays, patients with NSCLC are treated with chemotherapy, whereas advanced NSCLC treatment is based on combination chemotherapy, radiotherapy and/or surgery [3]. However, cancer recurrence is frequently observed [4].

Paclitaxel (PTX) is the main first-line chemotherapeutic drug used for the treatment of advanced NSCLC [5]. PTX, an anti-microtubule cytotoxic drug, inflicts severe untoward toxic effects, including neutropenia and peripheral neuropathy, due to the lack of specificity, and low water-solubility requiring the use of surfactant-solvent mixtures, like Cremophor EL:ethanol (50/50, *v*/*v*). Cremophor EL causes significant side effects including severe anaphylactoid hypersensitivity, hyperlipidemia, aggregation of erythrocytes, and neuropathy [6,7].

Targeted drug delivery is an important strategy to overcome these drawbacks. Hydrophobic chemotherapeutics including PTX, can be encapsulated, in polymeric nanoparticles (NPs) for example, to enhance dispersibility. Strategies include passive and active targeting. Passive targeting is based on the “enhanced permeability and retention” (EPR) effect, leading to NPs accumulation in the tumor [8], whereas, active targeting utilizes selective ligand-decorated NPs which undergo internalization into target cancer cells [6]. Often, passive and active targeting cooperate.

Recent active targeting strategies employ selective ligands including aptamers (APTs), short peptides, and small molecules [9,10,11]. APTs are an emerging class of promising targeting ligands for both cancer diagnostics and therapeutics [12]. S15-APT is an 85-base-long single-stranded DNA, selectively binding to NSCLC cells [13,14]. APTs possess several desirable qualities over other targeting strategies, mainly their tailorability to any target, small size, low immunogenicity, and high specificity [15].

However, when encapsulating a hydrophobic drug within NPs, the major frequent impediments are insufficient encapsulation efficiency (EE), low encapsulation capacity (EC), and premature drug release, which may cause undesired side effects [16]. Therefore, it is of paramount importance to develop NPs with proper features. Notably, NPs with an oily core possess important advantages for the overcoming of these limitations by stabilizing the lipophilic compounds and providing a hydrophobic environment in the inner core of NPs [17,18,19,20].

Herein, jasmine oil (JO; Log P = 3.10–3.32) [21] was selected to serve as the oily core for PTX coencapsulation in NPs, because of its good compatibility with PTX, since JO and PTX share a terpenoid structure and similar log P values (PTX Log P = 3.96) [22]. We hypothesized that the coencapsulation of PTX with JO would increase the physicochemical stability of PTX in aqueous media. Therefore, we studied the coencapsulation of JO with PTX, to improve the encapsulation and controlled release performance. JO is known for its anticancer, anti-inflammatory, antidepressant, antiseptic, antispasmodic, and sedative properties [23,24,25]. JO is extracted by solvents (like hexane) [25] and it contains 14% diterpenes, 5% triterpenes, and 42% esters, among which are monoterpene-esters (e.g., linalool and linalyl acetate) [26].

We have previously developed a targeted delivery platform against NSCLC cells for hydrophobic chemotherapeutics (e.g., PTX), based on PEG-PCL (PP) micelles, and decorated them with APTs as the targeting ligands [14,27]. The aim of the current study was to develop a cohesive JO-based oily core for the entrapment of PTX in order to obtain superior NP characteristics (size, EE, EC, drug retention, and cytotoxicity) to outperform currently existing targeted drug delivery systems. We found that co-encapsulation of PTX and JO within NSCLC-targeted APT-decorated NPs substantially reduced drug doses required for a cytotoxic effect. This may significantly reduce adverse side effects of PTX, as early release and exposure of healthy tissues would be avoided.

## 2. Materials and Methods

### 2.1. Materials

JO (*Jasminum officinale grandiflorum*) code: 1227000G, was kindly provided by Petrus Chemicals (Herzliya, Israel). Carboxy-polyethyleneglycol (COOH-PEG) (PEG-5kDa): polycaprolactone (PCL) (2.5 kDa) block-copolymer was custom-synthesized by Creative PEGWorks, (Durham, NC, USA). Amino-Cy5-labeled S15-APTs (nucleotide sequence: 5′-NH2-Cy5-ACG-CTC-GGA-TGC-CAC-TAC-AGG-CTA-TCT-TAT-GGA-AAT-TTC-GTG-TAG-GGT-TTG-GTG-TGG-CGG-GGC-TAC-TCA-TGG-ACG-TGC-TGG-TGA-C-3′ [13]), were purchased from BioSpring Biotechnology GmbH, (Frankfurt, Germany).

### 2.2. Methods

#### 2.2.1. Preparation of PEG-PCL NPs Containing JO and PTX

Polymetric NPs containing both JO and PTX (NPs/PTX-JO) and NPs lacking JO (NPs/PTX) were prepared by (low MW)-surfactant-free nanoprecipitation [28]. Different amounts of JO (1.5, 4.8, 9.6, and 19.2 μL) were dissolved in 0.8 mL acetonitrile (ACN) containing 70 μM PP and 14 μM PTX. Thereafter, ACN was added dropwise into ultrapure water (Biological Industries, Kibbutz Beit-HaEmek, Israel), and stirred at room temperature to obtain NPs with different PTX:JO molar ratios of 1:0.16 (1:1 *w*/*w* [29]),1:0.5, 1:1, and 1:2, respectively. S15-APTs were covalently conjugated to the NPs as previously described [14].

#### 2.2.2. Particle Size-Distribution Analyses

Particle size-distribution of the NPs was determined by dynamic light scattering (DLS), using a DLS analyzer (NICOMP^TM^ Particle Sizing System (PSS), Inc., Santa Barbara, CA, USA), as previously described [14,30]. The samples with different formulations were prepared as described in Section 2.2.1.

#### 2.2.3. Cryo-TEM Imaging

Aptamer-decorated nanoparticles (APT-NPs) encapsulating 14 µM PTX with either 88 µM or 176 µM JO, or in the absence of JO, in ultra-pure water, were analyzed by Cryo-TEM imaging using FEI Talos 200 C electron microscope (accelerating voltage 200 kV) at low dose imaging mode. Specimen preparation was as previously described [31,32]. Images were acquired by a FEI Falcon III direct-imaging camera, using “phase-plates” (FEI), to enhance image contrast [33].

#### 2.2.4. Analysis of Drug Encapsulation Capacity (EC) and Efficiency (EE)

To evaluate JO as an adhesive oily core aimed at enhancing PTX entrapment within the NPs, the amount of encapsulated drug (EC, µg PTX/mg polymer) within PP NPs and the EE (%) of PTX were quantified. Samples (NPs/PTX-JO) comprising 88 μM JO, 70 μM PP (0.525 mg/mL), and containing PTX at increasing drug:PP molar ratios, were prepared. Samples (NPs/PTX) of 70 μM PP with increased PTX content were prepared separately in the same manner. PTX quantification was performed as previously described [14,34].

#### 2.2.5. In Vitro Drug Release

The release profiles of PTX co-encapsulated with JO (1:1 *w*/*w*) from APT-NPs, vs. free PTX were investigated using dialysis under sink conditions as previously described [14,35]. Briefly, 1 mL PTX and JO-loaded APT-NPs or PTX-loaded APT-NPs were placed in 3.5 kDa MWCO dialysis bags (Sigma-Aldrich, Merck, Rehovot, Israel), submerged in 30 mL 10 mM phosphate buffer, pH 7.4, containing Tween 80 (0.1% wt) at 37 °C shaken gently. External buffer samples (30 mL) were drawn periodically, and the external solution was replenished with same volume of fresh buffer. Samples from each time point were freeze-dried, dissolved in ACN, and PTX was quantified by HPLC. Means of three independent experiments ± SE are reported.

#### 2.2.6. Cytotoxicity Assays

Human NSCLC A549 cells (ATCC) were cultured as previously described [14]. Our cell lines are routinely checked for mycoplasma using EZ-PCR™ Mycoplasma Detection Kit (Biological Industries, Kibbutz Beit-HaEmek, Israel). The effect of JO on selective cytotoxicity of PTX entrapped within APT-NPs was studied in A549 cells. Twenty-four hours before the experiment, A549 cells were seeded at a density of 0.6 × 10^4^ cells/mL in 96-well plates. To simulate the conditions in the human body, the APT-NPs encapsulating PTX with or without JO had undergone 24 h release (Section 2.2.5) prior to cell exposure to the NPs. To avoid cell exposure to Tween 80, 10% bovine serum albumin was used to capture the released PTX.

A549 cells were exposed to the APT-NPs/PTX at increasing PTX concentrations (0.1 nM–0.1 mM) for 2 h. Cells were also exposed to free PTX at increasing concentrations (0.1 nM–0.1 mM), for 2 and 72 h. Free PTX was dissolved in ACN (30 mM stock solution), such that 0.1% ACN was the highest concentration the cells were exposed to. Following 2 h treatments, an additional 72 h incubation in growth medium was performed to allow the drug to exert its cytotoxic activity. Cell growth inhibition was determined using a colorimetric XTT-cell proliferation assay, as previously described [14]. The Chou–Talalay combination index (CI) was used to examine the combined effect of drugs. The CI value was calculated by Equation (1) [36]:

(1) $$CI=\left(\frac{C\left(PTX\right)}{IC_{50\left(PTX\right)}}\right)+\left(\frac{C\left(JO\right)}{IC_{50\left(JO\right)}}\right)$$

where **IC_50(PTX)_** and **IC_50(JO)_** stand for the **IC_50_** values of **PTX** in the absence of **JO**, and the **IC_50_** of **JO** in the absence of **PTX**, respectively. Furthermore, exposure to (C(PTX)+C(JO)) induced 50% growth inhibition.

Herein we define a “synergy index” (**SI**) which is the inverse of the combination index, as defined in Equation (2):

(2) $$SI\equiv \frac{1}{CI}$$

such that **SI** > 1 indicates synergy between two drugs, **SI** = 1 indicates additivity, and **SI** < 1 indicates antagonism.

## 3. Results

### 3.1. Entrapment of PTX and JO within PEG-PCL NPs

**Figure 1:**
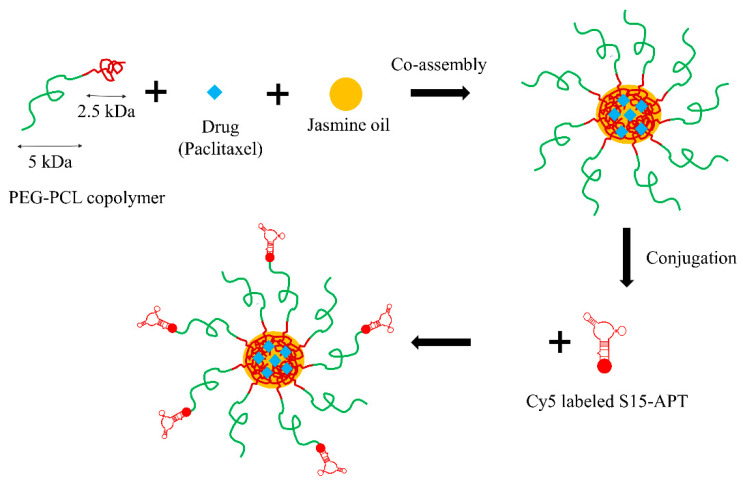
Schematic structure of the APT-NPs co-encapsulating JO and PTX.

The current nanosized delivery system comprises a biocompatible block-copolymer composed of PEG and PCL (PEG-PCL 5 kDa:2.5 kDa). Self-assembled micelles were prepared by the surfactant-free nanoprecipitation method [28]. These PP micelles coencapsulated JO and PTX in their hydrophobic core. The PP micelles were then decorated with S15-APT, an NSCLC-specific aptamer, as the targeting ligand, and were labeled with a diagnostic fluorescent tracer, Cy5 (Figure 1).

### 3.2. Size Distribution of NPs

**Figure 2:**
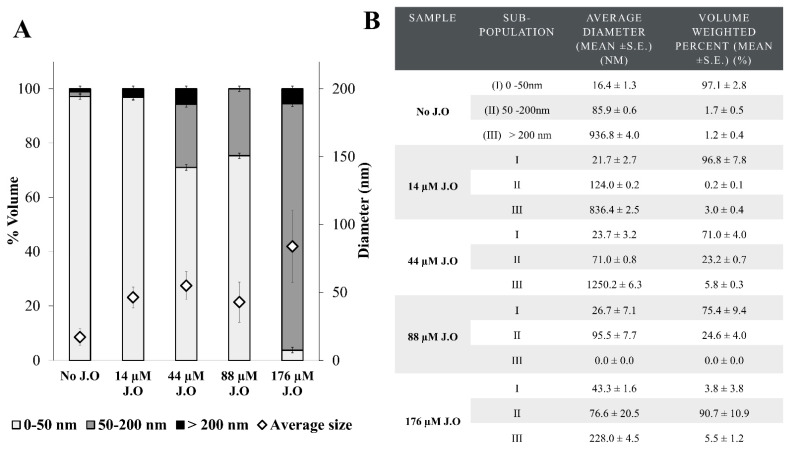
Volume-weighted size distribution of PEG-PCL/PTX NPs with different concentrations of JO determined using dynamic light scattering (DLS). PTX concentration = 14 µM, PP concentration = 70 µM. (**A**) Volume-weighted size distribution and average diameters (error bars represent the standard error of the measurements, based on duplicates); (**B**) characterization of subpopulations.

To prevent premature drug release, PTX was entrapped in the hydrophobic JO-core of PP NPs. The diameter of the JO-containing NPs was determined using DLS at several concentrations of JO. We aimed to obtain NPs with a diameter < 50 nm, to allow for an efficient receptor-mediated endocytosis (RME), as we have previously shown [14,27]. The DLS results demonstrated that the maximal concentration of JO that could be encapsulated in the PP NPs with an average diameter < 50 nm was 88 µM (Figure 2A). At this JO concentration, two populations of NPs were detected: 75 ± 4% of the NPs displayed an average size of 26.7 ± 7.1 nm, whereas the remaining 25 ± 4% had an average size of 95.5 ± 7.7 nm (Figure 2B). The most suitable composition selected for further experiments was 70:14:88 µM PP:PTX:JO.

### 3.3. Cryogenic-Transmission Electron Microscopy (Cryo-TEM) Analysis

**Figure 3:**
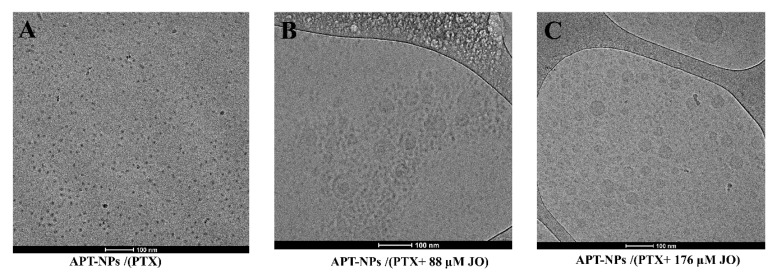
Cryo-TEM images of (**A**) APT-PP-NPs encapsulating 14 µM PTX; (**B**) APT-PP-NPs encapsulating 14 µM PTX + 88 µM JO; (**C**) APT-NPs encapsulating 14 µM PTX + 176 µM JO.

Cryo-TEM analysis revealed the morphology of the NPs: Aptamer decorated PP-NPs encapsulating 14 µM PTX (Figure 3A), were small (≈10 nm) simple spheres. The 14 µM PTX + 88 µM JO (Figure 3B), and 14 µM PTX + 176 µM JO (Figure 3C), demonstrated, in addition to NPs of ≈10–20 nm, clearly visible round core-shell structures of ≈50–100 nm.

### 3.4. Encapsulation Capacity (EC) and Encapsulation Efficiency (EE)

**Figure 4:**
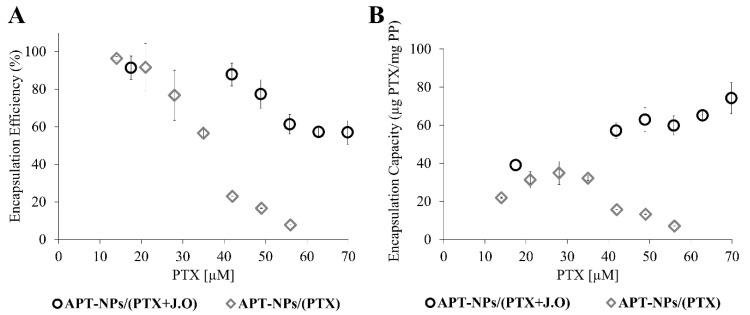
(**A**) Encapsulation efficiency (EE); (**B**) encapsulation capacity (EC) of PTX as a function of PTX concentration in the presence or absence of JO. Values presented are means ± SE.

The EC and EE of PTX were determined in the presence or absence of 88 µM JO (Figure 4). As shown in Figure 4A, the addition of JO markedly enhanced the EE of PTX. The maximal enhancement (3.8-fold) could be observed for 42 µM PTX, where the remarkable EE of PTX was 87.8% with JO, compared to only 23% without JO. Figure 4B shows that the addition of JO enhanced the amount of encapsulated PTX by 2-fold at the same PP concentration: the maximal EC increased from 35.0 ± 6.0 to 74.2 ± 8.2 µg PTX/mg PP. For PTX encapsulated in the absence of JO, at high drug concentrations, aggregation of free PTX was observed, as previously described [34,37,38], which explains the decrease in EC above the maximal value.

### 3.5. In Vitro Drug Release

**Figure 5:**
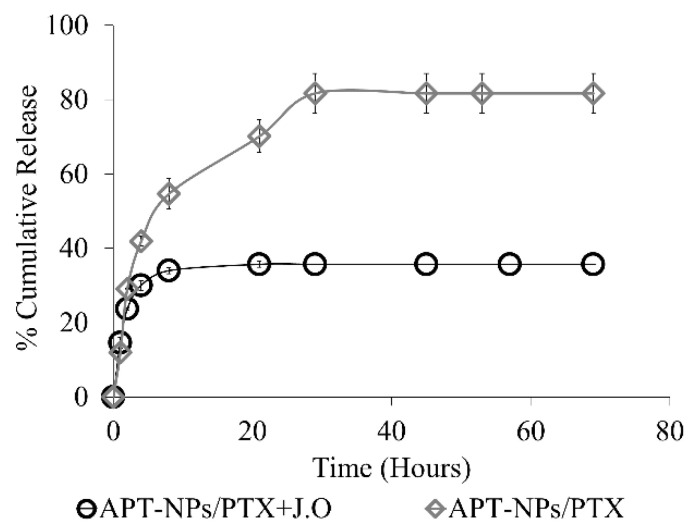
Drug release kinetics of 14 µM PTX from APT-NPs with and without 88 µM JO.

Drug release kinetics in the presence and absence of JO were determined over 69 h (Figure 5). The presence of JO within the core of the NPs suppressed PTX release. During early incubation times (up to ≈8 h), a burst of PTX release was apparent in both systems, while NPs lacking JO displayed a continuous release of the drug, which was apparently not tightly bound, at a somewhat slower rate, up to ≈28 h. In contrast, right after the initial burst release, JO-containing NPs attained a plateau without any additional release of PTX. As much as 64.3% of total PTX remained encapsulated within the JO-NPs during the 69 h of incubation, compared to only 18.3% PTX remaining in the JO-free NPs.

### 3.6. Cytotoxicity of PTX-Loaded APT-NPs against A549 Cells

**Figure 6:**
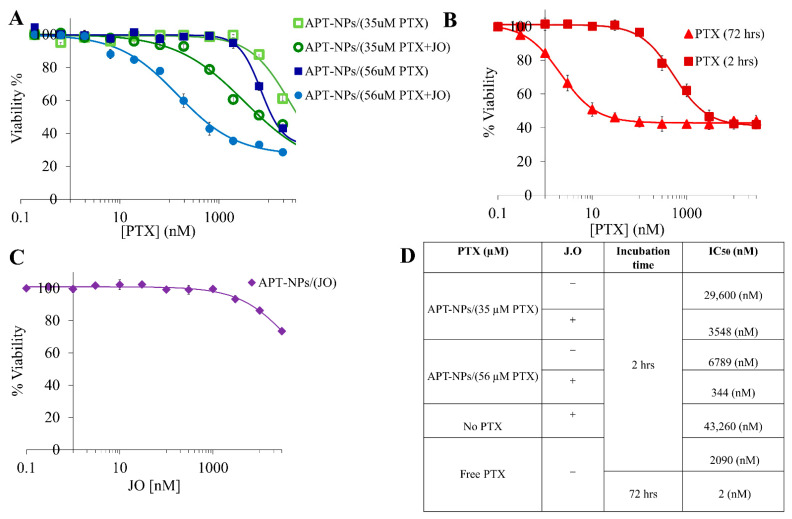
A549 cell viability as a function of PTX or JO concentration for (**A**) APT-NPs entrapping PTX (35 and 56 µM during drug loading) with or without JO; (**B**) free PTX was added to A549 cells for 2 or 72 h; (**C**) APT-NPs entrapping 88 µM JO. Values presented are means ± SE. Sigmoidal model curves were fitted using OriginPro 9.0 [14]; (**D**) IC_50_ values derived from the fitted dose–response curves; all *p*-values obtained were <0.05.

The cytotoxicity of PTX-loaded APT-NPs, containing or lacking JO, towards A549 cells was studied (Figure 6A). PTX was encapsulated within the APT-NPs at two concentrations: 35 and 56 µM. For each PTX concentration, the effect of JO addition was evaluated. The cytotoxicity combination index (CI) (Equation (1), Section 2.2.6) was calculated to determine the effect of the combination of PTX and JO. Furthermore, we defined a synergy index (SI) (Equation (2)) and used it to determine possible synergy between PTX and JO. For 35 µM PTX with JO, the calculated SI value was 3, whereas that for 56 µM PTX with JO, was 16.7; i.e., for both systems, a prominent synergistic effect between PTX and JO was observed, and the synergy was much greater at the higher concentration of PTX.

For the APT-NPs system encapsulating 35 µM PTX and 88 µM JO, the IC_50_ value after a 2 h exposure of A549 cells was 3.5 µM, whereas for the same concentration of PTX in the absence of JO, the IC_50_ value was 8.3-fold higher: 29.6 µM (Figure 6A,D). For the APT-NPs system containing 56 µM PTX and 88 µM JO, the PTX IC_50_ value obtained after a 2 h exposure of A549 cells was merely 0.3 µM, whereas in the absence of JO it was 6.8 µM; i.e., the presence of JO decreased the IC_50_ by nearly 20-fold (Figure 6D).

Remarkably, when APT-NPs co-encapsulating 56 µM PTX and 88 µM JO were compared to free PTX (upon 2 h of drug exposure), the IC_50_ value was reduced by 5.8-fold. Hence, these JO-containing PTX-encapsulated APT-NPs, killed NSCLC cells very efficiently and were much more potent than free PTX. We also tested the effect of 2 h vs. 72 h incubation of nonencapsulated PTX on A549 cells. We observed a three-orders-of-magnitude increase in the IC_50_ values, from the nM to µM range, when the exposure time was decreased from 72 to 2 h, demonstrating the significance of the incubation time on the cytotoxic capacity of PTX (Figure 6B,D).

The cytotoxicity of drug-free APT-NPs encapsulating solely JO was evaluated. The IC_50_ value obtained after a 2 h exposure of A549 cells was 43.3 µM (Figure 6C,D). We have previously shown that APTs and nonloaded NPs alone were nontoxic to A549 cells [14]. Significant differences were obtained between all IC_50_ values with *p*-value < 0.05, as determined by Student’s *t*-test.

## 4. Discussion

Despite its substantial side effects, PTX remains a dominant first-line anchor drug for the treatment of NSCLC and other malignancies [5]. Even targeted delivery systems often induce untoward toxic effects due to premature drug release, or low selectivity. To overcome these adverse side effects, we developed an aptamer-based, selectively targeted, delivery system harboring PTX, with enhanced entrapment and drug retention. In this respect, we have previously studied the S15 aptamer as a ligand for targeting NSCLC cells [27], and PP NPs as a polymeric drug delivery system [14]. We have demonstrated that the S15-APT ligand enables specific binding and uptake of the NPs by A549 cells; we have also studied six different human cell lines in terms of cellular internalization and cytotoxicity [26,27]. The internalization of S15-APT-NPs by target A549 cells was compared to that of normal human bronchial epithelial BEAS2B, cervical carcinoma HeLa, colon adenocarcinoma CaCo-2, neonatal foreskin fibroblast FSE, and human embryonic kidney HEK-293 cells. In that study we clearly established the high selectivity and specific binding of S15-APT-NPs to A549 cells followed by their efficient internalization. These results demonstrated 2–5 orders of magnitude higher selective cytotoxicity towards NSCLCs compared to the other cell type, reflecting a potentially outstanding therapeutic window [14].

In the present research, we developed a targeted delivery system that co-encapsulates a hydrophobic chemotherapeutic drug and JO, a terpenoid oil. We investigated the impact of JO, incorporated in the core of NPs as an adhesive solvent that inhibits PTX crystallization [32] and premature PTX release. We discovered that JO co-encapsulated in the core of the NPs, enhanced the EE from 23.0% to 87.8%, and the EC from 35.0 ± 6 to 74.0 ± 8 µg PTX/mg PEG-PCL. Hence, this constitutes the formation of an improved delivery system, which efficiently encapsulates a markedly increased drug payload. Next, premature drug release was circumvented as we demonstrated a significant increase in the percent of drug retained within the core of the NPs from 18.3% to 65% after 69 h of dialysis in buffer under sink conditions. Moreover, the NPs retained a small size < 50 nm, thereby facilitating efficient internalization via receptor-mediated endocytosis, as we have previously established [27]. The cytotoxicity of these NPs outperformed JO-free NPs by 20-fold and most importantly demonstrated a 6-fold more potent tumor cell kill than the free drug administered today in the clinic. The combination of PTX and JO displayed a synergistic cytotoxic effect. Thus, this nanomedicine delivery system holds great promise as an efficient platform to efficiently encapsulate hydrophobic chemotherapeutics, and has the ability to exert a potent cytotoxic effect on NSCLC cells.

In the absence of JO, many small simple spherical NPs (≈10 nm) were formed (Figure 2 and Figure 3A), while co-encapsulation with JO led to the formation of larger (50–100 nm), clearly core-shell structured NPs (Figure 3B,C), along with smaller NPs (≈10–20 nm). JO was responsible for this decorated core-shell nanodroplet morphology, which facilitated the entrapment of the drug cohesively in a liquid form. The selected JO concentration of 88 µM resulted in APT-NPs with an average diameter ≤ 50 nm. The suitable size for systemic drug delivery systems for passive and active targeting, is in the range of 10–200 nm. The latter upper limit is due to the effect of filtration in the spleen, not due to the gap size in tumor vasculature, which may be as large as 780 nm [39,40]. The ideal NP size for active uptake via RME is ≈10–50 nm [41]. Other oil-core systems (both targeted and nontargeted) possess an average diameter >100 nm [42,43,44,45,46], a disadvantageous size for active uptake via RME. It has been shown that NPs with an average diameter < 100 nm exhibit a significantly increased cellular uptake [41,47]. Moreover, as the size of NPs decreases, the uptake rate increases [48]. Herein, we developed an improved drug delivery system while maintaining the ideal average particle size (<50 nm) required for efficient uptake via RME. Endocytosis-mediated uptake may possibly bypass mechanisms of multidrug resistance (MDR) [49,50,51,52]. MDR to anticancer drugs via drug efflux transporters, frequently mediated by P-glycoprotein, continues to be a major impediment towards curative cancer therapy [53,54,55,56,57]. Hence, the uptake of targeted NPs into tumor cells via RME is of great importance towards the development of an efficacious cancer treatment modality [58].

Herein we overcame the problem of low EE reported previously for PTX systems [59,60]. The EE values obtained were slightly improved compared to the 47–80% reported in a previous study that co-encapsulated oils with PTX [42]. Moreover, the inclusion of JO in the core of APT-NPs, prevented most of the early drug release and facilitated the stabilization of PTX within the NPs (Figure 5). A different study co-encapsulating an antifungal agent with oil resulted in 65% drug release within 48 h [45], compared to only 35% PTX released during 69 h from our JO-containing APT-NPs. Evidently, APT-NPs containing JO, display a better sustained drug release profile than other oil-core NP systems. Furthermore, it should be noted that the only two previous studies co-encapsulating PTX did not use a terpenoid oil, and were nontargeted delivery systems [42,43].

The cytotoxic activity of APT-NPs was evaluated for two PTX concentrations, based on the results in Figure 4A, where the largest difference between the EE of the two systems was observed. PTX concentrations of 35 and 56 µM were selected. For each PTX concentration, the impact of JO addition was evaluated following a 24 h release experiment. For both systems, a synergistic effect between PTX and JO was observed, as the calculated SI values were impressive: 3 and 16.7, for 35 and 56 µM PTX, respectively. Borges et al., have also calculated the CI for the co-encapsulation of DOX and the bicyclic diterpene alcohol sclareol in human breast cancer cells. The CI obtained for the IC_50_ value was ≈0.35 [61], implying a synergistic effect with an SI value of 2.85. It can be concluded that the SI values we obtained were higher, therefore the combination of PTX and JO is more synergistic.

A remarkable result we obtained was the 5.8-fold lower IC_50_ value observed for 56 µM PTX in the presence of JO compared to free PTX. This was not the case for previously published delivery systems targeting A549 cells, where the free drug was more potent than the NPs entrapping PTX [62,63]. Additional major findings obtained herein were the 8.3-fold and 20-fold lower IC_50_ values seen for 35 and 56 µM PTX, respectively, in the presence of JO (Figure 6A,D). These findings outperformed a previous study showing that when PTX-OA is co-encapsulated with *Brucea javanica* oil, the IC_50_ value decreased by 2-fold, relative to oil-free PTX-OA. The study also revealed that as the incubation time was increased from 24 to 48 h, the IC_50_ values expectedly decreased [43]. We also demonstrated this phenomenon in Figure 6B; when the incubation time with free PTX was increased from 2 to 72 h, a three-orders-of-magnitude decrease in the IC_50_ values was observed. The IC_50_ value (2 nM) obtained here after 72 h incubation, agrees with the value of 3–4 nM reported in previous studies [64,65]. The IC_50_ value obtained here for A549 cells following a 2 h pulse incubation with JO encapsulated in APT-NPs was 43.3 µM (Figure 6D). This latter value is ≈4-fold lower than that (181 µM (0.025 mg/mL)) reported in a recent study showing that JO has an anticancer activity against MCF-7 breast cancer cells, where a 24 h incubation was used [23], suggesting that a much higher IC_50_ value would have been found for a 2 h pulse incubation. Moreover, compared to a study conducted on A549 cells, incubated with free JO for 72 h, where the JO IC_50_ value obtained was 0.012% (*v*/*v*), i.e. 835 µM [66], our delivery system achieved an almost 20-fold lower IC_50_ value, when solely loaded with JO.

The targeted nano-delivery system we developed herein co-encapsulating the highly compatible PTX and JO, exceeds other oil-core nano-systems and can therefore significantly improve drug delivery in general, and NSCLC treatment in particular. The JO core stabilized the drug within the NPs, thereby substantially reduced the drug doses needed for a cytotoxic effect. JO also reduced premature drug release, implying the minimization of deleterious side effects of PTX. Moreover, the small size of the NPs can enhance their efficient entry into cells via RME, as we have previously demonstrated [14,27], and may potentially bypass MDR efflux pumps. Furthermore, cytotoxicity studies showed a very potent activity when combining PTX with JO. Importantly, co-encapsulation following the example of PTX and JO may be widely applied to other targeted or nontargeted delivery systems. Furthermore, the APTs decorating the NPs can be easily substituted, therefore, this modular system can be implemented against any cancer type using proper APTs and hydrophobic anticancer drug combinations.

## 5. Conclusions

In the current study, a targeted drug delivery system that could potentially enhance the efficacy of NSCLC treatment was developed. The co-encapsulation of PTX and JO within APT-decorated PP NPs, specifically targeting NSCLC cells, was shown to substantially reduce PTX drug doses required for a therapeutic effect. We demonstrated that JO in the core of NPs enhanced both EE and EC of PTX, while diminishing premature drug release, resulting in a synergistically enhanced cytotoxic activity. The improved performance of this targeted delivery system and the reduced PTX drug doses are crucial to circumvent the major side effects of PTX, which inflicts neutropenia, leukocytopenia, thrombocytopenia, anemia, and peripheral neuropathy [67,68]. Moreover, this nanomedicine delivery system retained a small mean NP size of <50 nm, suitable for the uptake via RME, thereby, potentially evading MDR efflux pumps residing in the plasma membrane, such as P-gp, which are major obstacles to curative chemotherapy [40,49,50,51,52]. These findings demonstrate high compatibility and remarkable cytotoxic synergy between PTX and JO, complementing the high selectivity of the APT-NPs we have previously established. The contribution of JO to the physiochemical stability of PTX-loaded NPs in aqueous solutions may be widely applicable to different PTX delivery systems. Although the efficacy of this targeted delivery system was demonstrated against NSCLC cells as a model tumor cell line, it can be widely applied to any tumor cell target of interest, as APTs can be easily tailored and adapted to selectively bind to and enter various malignant cells. Hence, the currently developed delivery system offers versatile applicability, high efficacy, and potential personalization of the targeting moiety for precision medicine.

## Data Availability

The data presented in this study are available on request from the corresponding author.
